# Trustworthy Deep Feature Extraction and Ensemble‐Based Machine Learning Approach for Breast Cancer Detections

**DOI:** 10.1049/htl2.70062

**Published:** 2026-04-28

**Authors:** Md. Rashed, Mohammad Kamrul Hasan, Md. Imran Hossain, Md. Sarwar Hosain, Abdul Hadi Abd Rahman, Shayla Islam

**Affiliations:** ^1^ Department of Information and Communication Engineering Pabna University of Science and Technology Pabna Bangladesh; ^2^ Faculty of Information Science and Technology Universiti Kebangsaan Malaysia Bangi Selangor Malaysia; ^3^ Institute of Computer Science and Digital Innovation UCSI University Kuala Lumpur Malaysia

**Keywords:** breast cancer, deep learning, ensemble learning, machine learning, SVM, VGG‐16

## Abstract

Breast cancer (BC) has become a major public health concern and is critically associated with the highest global death rate for cancer detection. The diagnosis process and the techniques remain complex and often influenced by the diagnostician's background, which makes it challenging. Despite advancements in BC detection, existing methods cannot often effectively combine interpretability and high accuracy in complex imaging data, limiting their clinical applicability. This study proposes a reliable strategy for detecting BC by combining deep learning (DL) strengths with ensemble‐based machine learning (ML) techniques. ML models offer interpretability and generalisation, while DL enhances the ability to learn and uncover hidden patterns in complicated BC images. The pre‐trained model is used in the proposed technique for effective feature extraction, followed by applying eight different ML models to identify BC. The performance of the study is evaluated in terms of precision, recall, F1‐score, and confusion matrices for all classifiers. In addition, ROC curves are drawn for each classifier. Our rigorous experimentation yields compelling results that demonstrate exceptional performance compared with those of existing state‐of‐the‐art models. We achieve a higher accuracy rate of 97.50%, a precision of 97.15%, a recall of 97.00%, and an F1‐score of 96.98%. Furthermore, we determine that the support vector classifier is the most effective ML model when integrated with the pre‐trained VGG‐16 architecture. The strategies, exhaustive performance analysis, and reliable assessment presented in this research provide valuable advances in BC detection, helping doctors make better decisions, offering better patient care, and improving BC outcomes.

## Graphical abstract

This study presents a trustworthy method that integrates machine learning and deep learning methods, using pre‐trained models to extract features and build a reliable prediction model. An innovative feature of this study is extracting fine features from images of breast cancer, which provides a trustworthy evaluation of the BC identification system's efficiency, scalability, availability, and dependability.

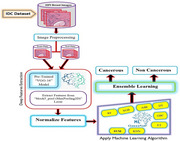

## 1 Introduction

### 1.1 Background Study

Globally, breast cancer (BC) is one of the critical diseases with a high death rate that occurs when abnormal cell proliferation in breast tissue symbolises the progression of malignancies. Statistics on global health indicate that the most prevalent cancer in women to be diagnosed is BC [1]. Over 2.26 million women will have new instances of BC in 2030. From statistics, the top 10 countries were listed where BC is most common in women with higher death rates, as presented in Table 1 [2]. Middle‐aged women are predominantly affected by breast tumours, which are regarded as a deadly illness.

**TABLE 1:** Highest rates of occurrence and deaths from breast cancer.

Rank	Country	Number	ASR/100,000
1	Belgium	11,734	113.20
2	The Netherlands	15,725	100.90
3	Luxembourg	497	99.80
4	France	58,083	99.10
5	New Caledonia	185	99.00
6	Denmark	5083	98.40
7	Australia	19,617	96.00
8	New Zealand	3660	93.00
9	Finland	5228	92.40
10	US	253,465	90.30

*Source*: Adapted from World Cancer Research Fund International, 2024.

Different methods have been developed for various technologies like image segmentation and DL models to classify the disease [3]. The use of histopathological images has been beneficial for both BC screening and treatment due to the phenotypic information. Deep neural networks (DNNs) are often used to detect BC with higher accuracy [4]. Researchers have presented that the critical first step in managing the side effects of BC is early diagnosis and treatment. Therefore, early diagnosis with accurate detection has been challenging [5]. Tumour lesions have been identified and localised on images in several investigations [6]. Automated extraction of higher‐level abstract information from an image is possible with the DL technique [7]. However, the features included in the data were retrieved using the multilayer perceptron network (MLP) approach. The network's structure was built to enable the analysis of how the data altered when the dimensions climbed or fell [8]. CNN is the most used deep learning (DL) method for image processing [9]. However, transformer‐based architectures have shown encouraging results in several computer vision tasks [10].

The FC and group convolution layers with a discrete cosine transform [11] construct a rotation‐invariant transition medium. This integration reduces the number of parameters and improves data efficiency by encoding and parameterising the model space, hence minimising data loss and computational costs. The diagnosis involves analysing histopathological images of cancer cells, a laborious process requiring skill.

**FIGURE 1:**
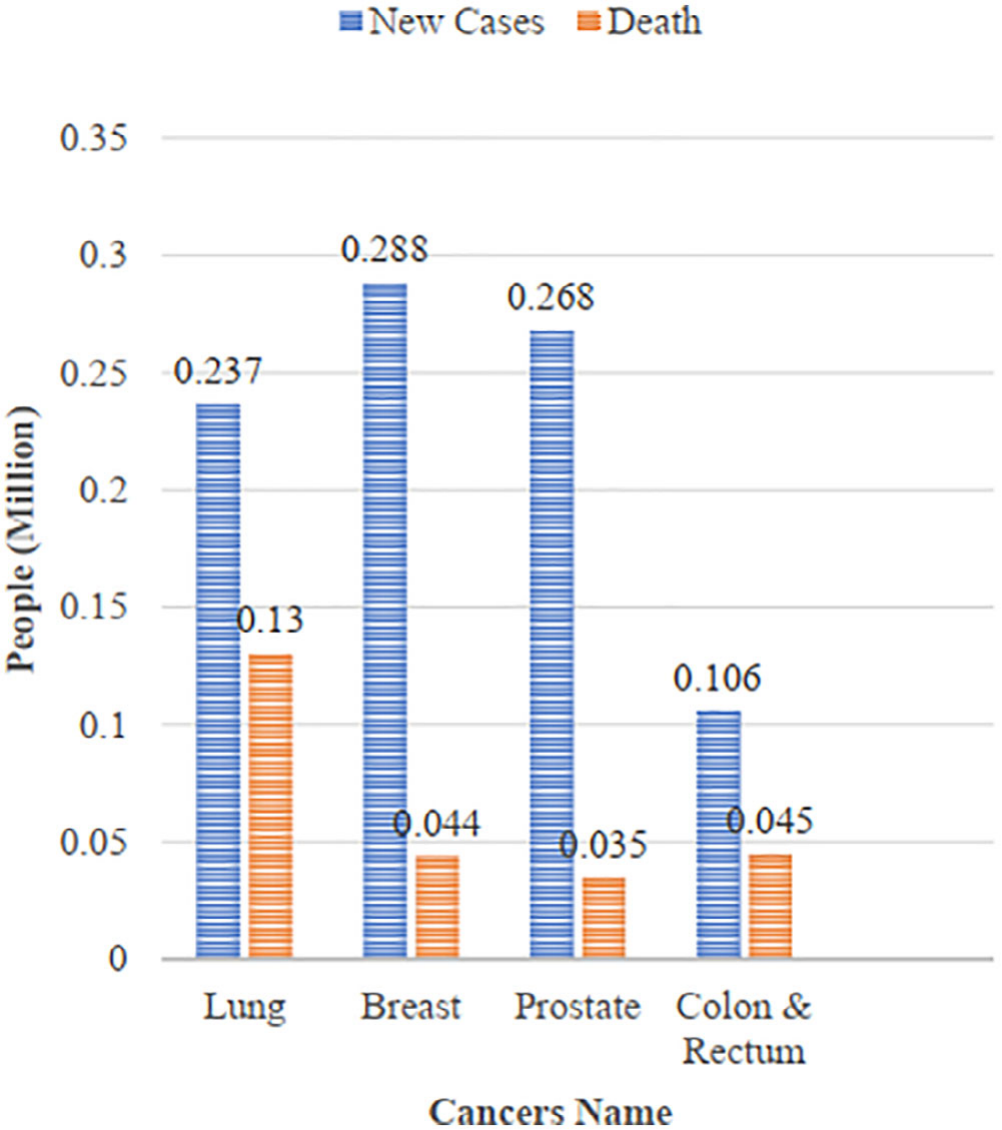
Statistics of cancer in 2022 (new cases and deaths).

The main function of image processing is feature extraction [12]. The World Health Organisation (WHO) cancer report for 2020 is published in [13, 14, 15], and Figure 1 depicts a projected statistical representation of several types of cancer in 2022. Numerous methods, such as computed tomography scans, ultrasonography, thermography, mammography, MRI scans, and histology, can be used to obtain medical images [16]. Eighty per cent of cases are of the most frequent kind, invasive ductal carcinoma (IDC), which attacks breast tissues nearby. The state of HER2 and hormone receptors can be used to categorise IDC. Ten to fifteen per cent of instances of BC are caused by invasive lobular carcinoma (ILC), which is harder to find with mammography and less prevalent than IDC [17]. Thermography, based on infrared photography, accurately measures surface temperatures, making it a useful predictor of breast tumours [18]. Reliable proof for BC prevention and treatment strategies remains elusive despite significant academic and medical efforts [19, –21] and an ensemble machine learning (ML) model can significantly improve the prediction with low time consumption [22].

### 1.2 Motivation of the Study

A novel ML model based on a metaheuristic algorithm and a truncated C‐means segmentation was proposed [23] to classify and detect BC from mammography images. As demonstrated by Prakash et al. [24], deep convolutional spiking neural networks enabled the automatic identification and categorisation of BC from mammography images.

However, these approaches often lack the integration of interpretability and fine‐grained feature extraction, which limits their efficacy. This study addresses these gaps by proposing a novel hybrid model that synergistically combines the robust feature extraction capabilities of the pre‐trained VGG‐16 DL model with the interpretability and generalisation of multiple ML techniques, offering a more accurate and clinically applicable diagnostic tool compared to existing methods.

Our research addresses these shortcomings by offering an improved model for BC diagnosis. By leveraging the strength of a pre‐trained “VGG‐16” DL model for effective feature extraction and integrating several ML techniques, including Ada Boost (AdB), K‐nearest neighbours (KNNs), gradient boosting classifier (GBC), decision tree (DT), random forest (RF), support vector classifier, extra trees (ET), and extreme gradient boosting (XGB), we propose a crossbreed and a reliable BC prediction model. This model combines the interpretability of ML techniques with the subtle pattern recognition of DL models, ensuring a robust diagnostic framework.

### 1.3 Contributions

This research study makes unambiguous contributions in the following ways:

1. The work presents a trustworthy method that integrates ML and DL methods, using pre‐trained models to extract features and build a reliable prediction model.
2. The study incorporates an extensive performance investigation, utilising stringent assessment measures to guarantee the proposed model's dependability, precision, and overall effectiveness.
3. An innovative feature of this study is extracting fine features from images of BC, which provides a trustworthy evaluation of the BC identification system's efficiency, scalability, availability, and dependability.
4. This research facilitates better‐informed decisions by medical professionals, enhancing patient care and ultimately leading to improved results for individuals with BC.

### 1.4 Outline

This paper is organised into some sections to provide a comprehensive overview of the proposed hybrid approach to BC classification. The introduction outlines the background of BC as a significant global health issue, the motivation behind the study, and its key contributions, including the integration of DL and ML techniques. Section 2 analyses prior research on BC classification, highlighting methods like transfer learning, CNNs, and ensemble models, while identifying gaps such as limitations in dataset diversity and scalability. Section 3 explains the systematic approach used, including dataset acquisition, preprocessing steps like image resizing and normalisation, deep feature extraction using the VGG‐16 pre‐trained model, and the application of ML algorithms like SVM, RF, and GBC. Section 4 estimates the model's performance using accuracy, precision, recall, and F1‐score, with SVM achieving the highest accuracy of 97.50%. It also discusses findings, performance robustness, and limitations, such as the focus on a single BC subtype and dataset variability. Finally, Section 5 summarises the contributions, emphasising the model's potential for clinical applications and suggesting directions like multimodal data integration and enhanced generalisation for real‐world usage.

## 2 Related Works

BC is a major global health anxiety, and early identification of cancerous cells is critical for successful diagnosis and effective treatment. Histopathological image examination and feature extraction play a pivotal role in image processing by permitting the recognition of important patterns and traits. ML algorithms have offered significant advancements in BC detection and analysis by introducing innovative techniques. For instance, Mahmud et al. [4] applied transfer learning models such as ResNet‐50, ResNet‐101, VGG‐16, and VGG‐19 to a dataset of 2453 histopathology images, classifying IDC and non‐IDC cases, with ResNet‐50 outperforming other models at 90.2% accuracy. However, their approach faced limitations in handling diverse datasets, which impacted the generalisability of the models. Ardavan Modarres et al. [10] integrated patch embedding from transformer‐based architectures with fully CNN‐based models, achieving high accuracy across varying magnifications (95.42%, 98.16%, 96.05%, and 97.92% for 40×, 100×, 200×, and 400×, respectively). Despite these promising results, the reliance on specific magnifications limits the adaptability of their model to heterogeneous datasets.

Similarly, Anjum et al. [12] employed feature extraction techniques such as histograms of oriented gradients (HOG) and Canny Edge detection, combined with feature reduction using principal component analysis, and trained their model using SVM, LR, and AdaBoost. While achieving 94.00% accuracy, their method struggled with scalability and computational efficiency when processing large datasets. Using a deep belief network (DBN), Hirra et al. [25] developed Pa‐DBN‐BC, a unique patch‐based DL technique for BC detection and classification. This study [26] introduces a DNN with mixed activation functions to classify BC using the WDBC dataset and ten‐fold cross‐validation. Their approach involved unsupervised pre‐training and fine‐tuning to extract features from image patches, achieving 86.00%.

This paper [27] compares several classical ML models for BC diagnosis using LASSO‐based feature selection, where RF achieved the best accuracy (90.68%), but lacks DL and explainability. Kulkarni and Sundaray [28] used a fold‐based training strategy with a pre‐trained residual feature extraction model to classify IDC datasets, achieving 91.00% accuracy. Their fold‐based strategy improved outcomes but revealed gaps in the comprehensive evaluation of large‐scale datasets. Lastly, Jin and Xie [29] developed a self‐defined CNN and compared its act to ResNet‐50. Despite achieving an accuracy of 86.57%, their study emphasised the difficulty of maintaining consistent performance across diverse data distributions. This study [30] classifies BC histopathological images using a hybrid dataset from BreakHis and Histo, employing transfer learning with pretrained models. DenseNet201 achieved 91.37% accuracy at 200× magnification. The research gap includes the need for validation on more diverse datasets and magnification levels. With less training time and parameters, this work [31] suggests a TokenMixer hybrid architecture that blocks CNNs and vision transformers (ViTs) to improve the categorisation of BC subtypes in histopathology images. The accuracy of the classification is 97.02% for binary and 93.29% for multiclass images. Nevertheless, it is not validated on various histopathological image types or real‐world circumstances, and it does not address energy efficiency or computing cost for deployment in resource‐constrained environments. These studies underscore the potential of advanced models like CNNs, transformer‐based architectures, and hybrid techniques in BC detection. However, limitations such as dependency on specific datasets, poor scalability, and challenges in generalising to heterogeneous data persist. Addressing these gaps through robust frameworks capable of handling diverse datasets is crucial to improving diagnostic accuracy and reliability. A recent study [32] evaluates multiple ML classifiers on the WBCD dataset and applies SHAP and LIME to explain RF predictions, achieving 99.46% accuracy, though the study is limited to a single dataset and classical ML models.

Many studies on BC detection use classical ML, DL, or hybrid models, but they often rely on single datasets, handcrafted features, or specific image magnifications, limiting generalisation and clinical applicability. Deep models can be computationally heavy and lack interpretability, and few works apply explainable AI. In contrast, our work combines deep feature extraction with ensemble‐based ML, offering better robustness, comparative evaluation, and explainability for reliable clinical use. Table 2 represents the summary of related works on BC detection.

**TABLE 2:** Summary of related works on breast cancer detection and classification.

Ref. No.	Work	Method	Performance result	Limitations
[4]	Breast cancer detection and classification	ResNet‐50, ResNet‐101, VGG‐16, VGG‐19	90.2% (ResNet‐50)	Limited generalisability due to dataset diversity
[10]	Breast cancer detection using transformer + CNN	Patch embedding + CNN	95.42%–98.16% (40×–400×)	Performance depends on specific magnifications; limited adaptability
[12]	Breast cancer detection using feature‐based ML	HOG + Canny + PCA; SVM, LR, AdaBoost	94.00%	Poor scalability and computational efficiency for large datasets
[25]	Patch‐based breast cancer detection	DBN, patch‐based learning	Not specified	Extensive training required; limited cross‐dataset evaluation
[26]	Breast cancer classification using DNN	Unsupervised pre‐training + fine‐tuning	86.00%	Moderate accuracy; lacks interpretability
[27]	Breast cancer diagnosis with classical ML	LR, KNN, RF, GB, XGB, MLP, SVM + LASSO	90.68% (RF)	Lacks deep learning and explainability
[28]	Breast cancer classification using fold‐based training	Pretrained CNN, fold‐based strategy	91.00%	Limited evaluation on large‐scale datasets
[29]	Breast cancer detection using custom CNN	Custom CNN vs ResNet‐50	86.57%	Inconsistent performance across diverse data
[30]	Breast cancer classification with transfer learning	DenseNet201	91.37% (200×)	Needs validation on diverse datasets and magnifications
[31]	BC subtype classification using TokenMixer	TokenMixer (CNN + ViT)	97.02% (binary), 93.29% (multi‐class)	Not validated on diverse images; ignores energy/computing costs
[32]	Breast cancer prediction using ensemble ML + XAI	RF, XGB, SVM, MLP, gradient boost + SHAP/LIME	99.46% (RF)	Limited to a single dataset; classical ML models only

## 3 Methodology

**FIGURE 2:**
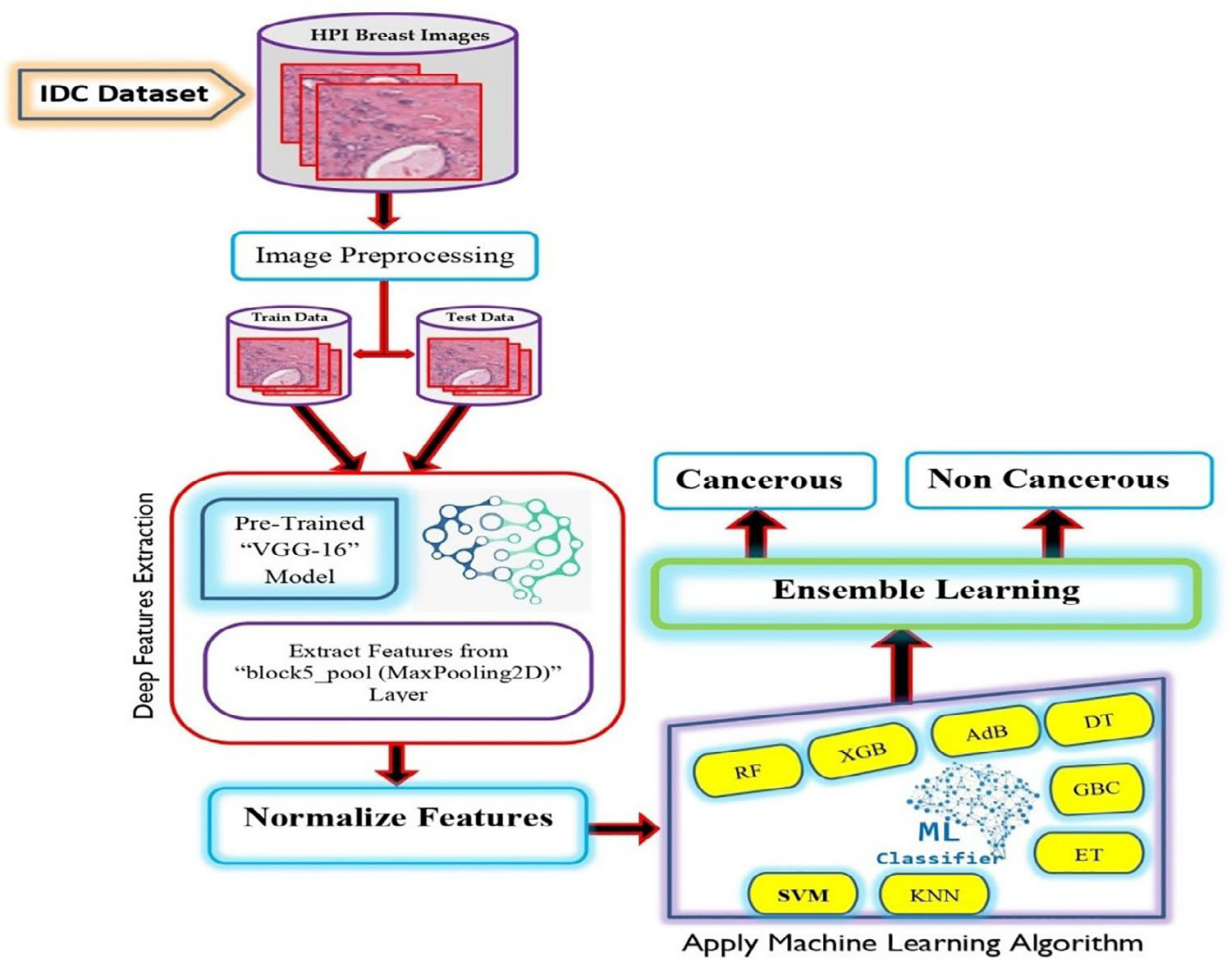
The architecture for the suggested approach to the detection of breast cancer.

This work employs a systematic approach to develop a reliable ML and deep feature extraction model for BC diagnosis. The methodology involves several key steps: acquiring a histopathological image‐based dataset, preprocessing the data with techniques like label encoding and image resizing, extracting deep features using the VGG‐16 pre‐trained model, and classifying these features with ensemble‐based ML models such as SVM, AdB, GBC, and RF. The final performance evaluation uses metrics like accuracy, precision, recall, and AUC–ROC to ensure robust and accurate BC detection. A detailed explanation of each step is provided below, and is illustrated in Figure 2.

### 3.1 Dataset Acquisition

In this stage, a suitable dataset for BC HPI must be found and chosen. It also entails getting the required authorisation. Data access and use require rigorous observance of ethical norms and laws, as well as privacy. This work used the IDC dataset, which is the maximum common subtype of breast tumours. Overall, 162 complete mount slide images of BC specimens scanned at 40× magnification make up the collection. The whole amount of the slides is devoted to IDC‐affected regions. Accurate detection of IDC locations across sample slides is crucial for automatically determining aggressiveness [33]. Specifically, the 2000 images in the HPI BC imaging dataset were employed in our investigations. Out of all the photos, 1000 were categorised as non‐cancerous cases with no evidence of IDC, whereas 1000 were classified as positive for IDC, signifying the existence of BC. The source [34] provides access to the IDC dataset, which is available to the public. It is an important tool for creating and testing automated techniques for detecting BC. These automated techniques have the potential to save pathologists time and lower errors in clinical practice by precisely recognising and classifying BC subtypes [35].To improve the BC detection procedure, (277, 524, 50 × 50) pixel patches were taken out of the original dataset. The research on the identification of BC uses these patches as input samples. 78,786 and 198,738 of the retrieved patches were categorised as IDC positive and negative, respectively [33].

The IDC dataset was selected for a number of reasons:

1. **Pertinence to the identification of breast cancer**: The dataset focuses on the most prevalent and dangerous kind of BC, IDC. Because the goal of our study is to increase the identification of BC, it is very pertinent to choose a dataset that focuses on this subtype.
2. **Public availability**: The openness and repeatability of our tests are guaranteed by the IDC dataset's public availability. The dataset is available to researchers worldwide, who may use it to confirm our findings.
3. **Large and diverse sample size**: The IDC dataset offers a significant and varied sample size of 2000 pictures and over 277,000 patches, allowing for a thorough assessment and broad application of our suggested methodology.
4. **Evaluation benchmark**: The IDC dataset, a commonly used dataset in the field, acts as a standard for techniques used to identify BC. It is possible to fairly assess the effectiveness of our strategy by contrasting our findings with previous research on the same dataset.
5. **Possible clinical impact**: Both clinical practice and patient outcomes may be greatly impacted by an accurate diagnosis of BC. Our study is to advance automated techniques that can help pathologists make prompt and precise diagnoses using the IDC dataset.

To ensure external validation of our proposed model, we additionally utilised the BreakHis dataset, which comprises 9109 RGB histopathological images of breast tumours from 82 patients. The images were acquired at four magnification levels (40×, 100×, 200×, and 400×), consisting of 2480 benign and 5429 malignant samples [36].

### 3.2 Data Preprocessing

Image preprocessing is essential to our BC detection technology, as it sets up the input images for further analysis. To improve the quality of the raw input data and extract useful information, a number of operations are applied to them. A thorough preprocessing of the images can greatly increase the accuracy and effectiveness of the analysis that follows. The recommended approach is used to prepare the HPI images for BC diagnosis by applying several image preprocessing techniques. The procedure is provided below briefly:

- **Image resizing**: To maintain a consistent input image size and reduce computing costs, we resized the images to a predetermined 224 × 224 pixel size. We may effectively use the features that the model has learned by resizing the images to be the same as the input shape of the model. Resizing to a standard size also improves training and inference computation performance, which raises overall process efficiency.
- **BGR to RGB conversion**: The initial colour space used for the HPI graphics was BGR. We changed them to the more widely known RGB colour scheme, which is more appropriate for applications involving image processing later on. This conversion process makes sure that colours are represented consistently throughout the dataset.
- **Image sharpening filter**: To bring out the small features and edges in the images, we used an image sharpening filter. By increasing the contrast between image characteristics, this filter helps with feature extraction by making them easier to differentiate from one another.
- **Image scaling**: To scale the image, we divided the pixel values by 255. This normalised the data and brought them into a predetermined range. This normalisation phase helps the following ML algorithms converge by maintaining the pixel values within a fixed numerical range.
- **Image labelling**: Textual object labels were present in the original HPI dataset. We carried out image tagging by translating the object labels into numeric labels in order to make the ensuing analysis easier. This translation makes a variety of ML algorithms usable.

**FIGURE 3:**
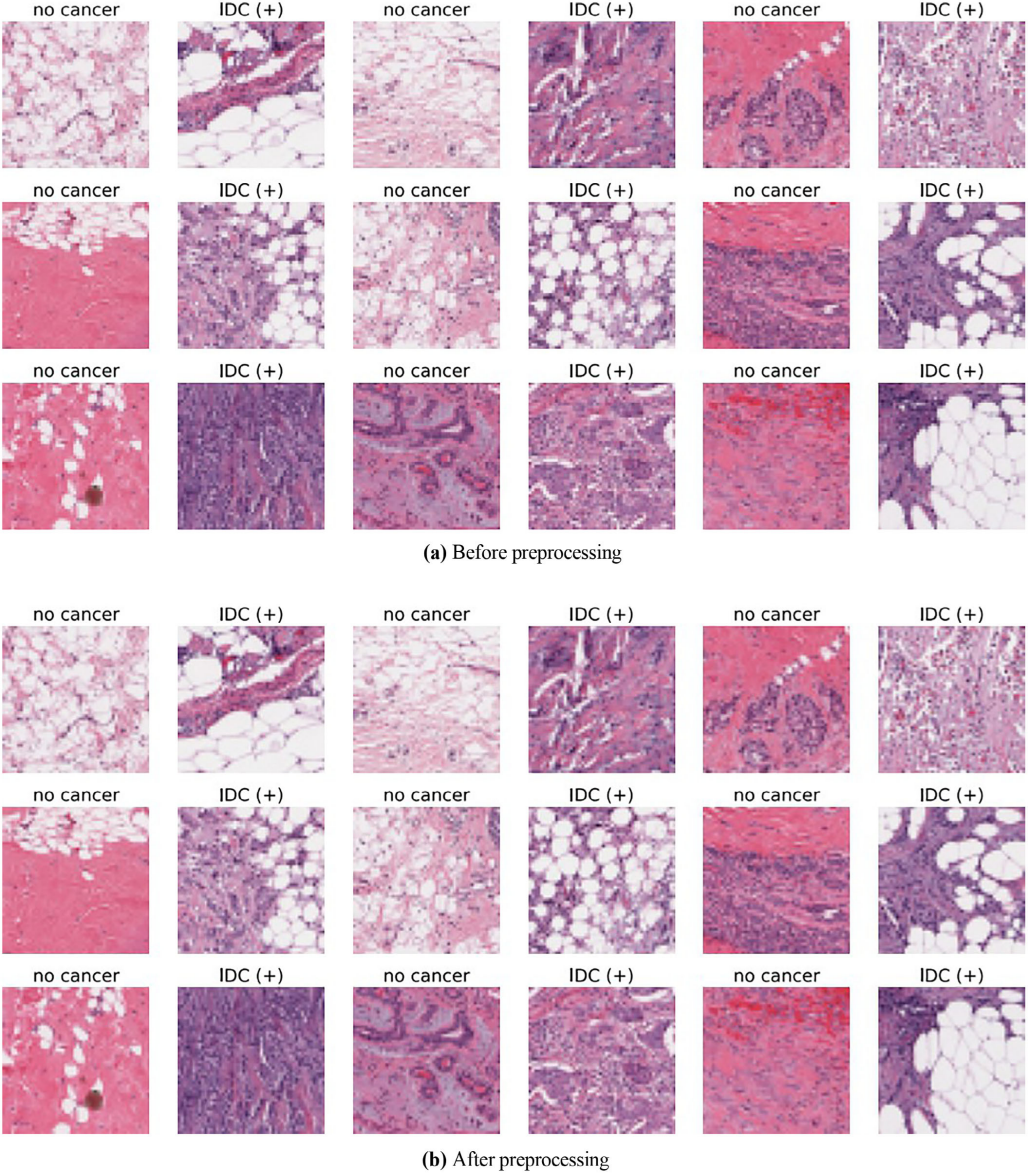
Sample images from the IDC dataset.

Overall, the preprocessing methods employed in our breast methodologies for detecting cancer have many advantages. To start, resizing images guarantees constant image dimensions, reducing bias caused by differences in dimensions. Second, standardising colour representation for trustworthy feature extraction is achieved by changing BGR to RGB colour space. Third, by enhancing tiny details, the image‐sharpening filter improves the ability to distinguish essential characteristics. Fourth, scaling helps with practical model training by normalising pixel values. Lastly, the application of various classification techniques is made possible by image labelling. By using these methods, we improve the precision and dependability of our system for detecting BC and get HPI images ready for ML and deep feature extraction. Figure 3 shows the sample images from the IDC dataset.

### 3.3 Extraction of Deep Features

**FIGURE 4:**
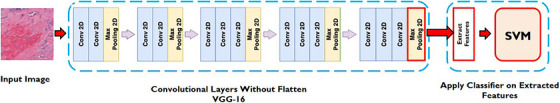
The architecture for the suggested approach to the detection of breast cancer.

The idea of deep feature extraction and how it is used to identify BC is explained in this part. Deep feature extraction is the procedure of employing a DNN to extract useful and instructive features from unprocessed data. We used the VGG‐16 architecture for deep feature extraction. To extract fine features from images of BC, the pre‐trained VGG‐16 architecture for deep feature extraction in BC classification includes multiple layers, specifically block5pool (MaxPooling2D), block4pool (MaxPooling2D), and block2pool (MaxPooling2D). The process starts with a Conv2D layer and iteratively detects and refines image features by adding layers of activation and batch normalisation. Dropout layers stop overfitting, while MaxPooling2D layers minimise spatial dimensions. In order to further refine and integrate the collected features, MaxPooling2D condenses the spatial dimensions into a single vector per feature map. This vector is then fed into a sequence of dense layers interspersed with activation, batch normalisation, and dropout layers. VGG16 can understand and represent the complex and hierarchical features of breast tissue with more accuracy because of this structured approach, which also makes it easier to differentiate between benign and malignant cancers. Figure 4 shows the VGG16 model architecture for deep feature extraction. The detailed procedure of deep feature extraction is presented in Algorithm 1.

**Convolution operation**: Given an input feature map *X* and a filter *W*, the convolution output *Y* at position (*i*,*j*) is

(1) $$\;Y_{i,j}={\left(X*W\right)}_{i,j}=\sum_{m=0}^{M-1}\sum_{n=0}^{N-1}X_{i+m,j+n}\cdot W_{m,n}\;$$

**Max pooling**:

(2)

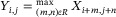

ALGORITHM 1 **Feature extraction using pretrained VGG‐16**

**Table:** 

**1**.	**Initialise**:
	Load the VGG‐16 model without a fully connected layer:
	*base_model ←* VGG16(weights = ‘imagenet’, include_top = False).
	Define the feature extraction layer:
	*layer_name ←* ’block5_pool’
	*model ←* Model (inputs = base_model.input,
	outputs = base_model.get_layer (layer_name).output)
**2**.	**Normalise**:
	*X ← X*/255
**3**.	**Feature Extraction**:
	*extracted_features ←* model.predict(X)
**4**.	**Flatten Features**:
	Reshape the features into a 2D array:
	*Features ←* reshape(*extracted_features*, (*N*, –1))
**5**.	**Save Features**:
	Save extracted features and corresponding labels:
	np.save(’train_features.npy’, Features)
	np.save(’Y_train.npy’, labels)
**6**.	**Return**:
	*Features*

### 3.4 Machine Learning Models

A combination of conventional ML methods and deep feature extraction was employed in the BC diagnosis investigation. This approach not only reduced dimensionality and corrected the class imbalance, but it also made use of model compression techniques, enhanced out‐of‐distribution detection, and permitted exploratory data analysis. In order to achieve these objectives, this study employed a range of ML methods, including ensemble‐based algorithms, which are described below:

- **Decision trees (DT)**: A structure that resembles a flowchart and is used for forecasting or decision‐making is called a DT. It consists of nodes representing attribute tests or judgements, branches displaying the outcomes of these tests or decisions, and leaf nodes displaying the decisions or conclusions drawn [37].
- **Support vector machine (SVM)**: SVM is a type of supervised ML method used for regression and classification purposes. Even yet, classification issues are the best application for regression problems. The basic purpose of the SVM approach is to discover the optimal hyperplane in an N‐dimensional space that will separate data points into discrete classes in the feature space [38].
- **AdaBoost (AdB)**: Adaptive boosting or AdaBoost is a potent ensemble ML method that is extensively used for regression and detection problems. Its main goal is to improve poor learner performance by integrating them into a reliable and precise ensemble model, usually basic models or classifiers [39].
- **Gradient boosting classifier (GBC)**: GBC is a potent enhancement technique that strengthens a lot of weak learners. Every new model is trained with the aim of minimising the loss function, which may be anything from the mean squared error to the cross‐entropy of the prior model. By computing the gradient of the loss function about the current ensemble's predictions, a new weak model is trained in each cycle [40].
- **K‐nearest neighbours (KNN)**: The KNN approach is a non‐parametric supervised learning classifier that uses proximity to classify or forecast a single data point's grouping. These days, it is one of the most popular and simple regression and classification classifiers in ML [41].
- **RF**: It can be used for ML problems that contain regression and classification. Its foundation is the concept of ensemble learning, which joins various classifiers to improve the model's functionality and discourse on a challenging issue [42].
- **XGBoost (XGB)**: XGBoost's architecture prioritises speed, simplicity of use, and performance on large datasets. Because it does not need parameter adjustment or optimisation, it may be used immediately after installation without requiring any extra settings [43].

### 3.5 Hyperparameter Optimisation

To ensure optimal performance of the ML models, we employed a grid search approach for hyperparameter optimisation across all classifiers, including SVM, AdB, GBC, RF, KNN, DT, ET, and XGB. Grid search was selected due to its systematic exploration of predefined hyperparameter combinations, guaranteeing thorough evaluation to identify the settings that maximise model accuracy and robustness. For SVM, we tuned the regularisation parameter C (range: [0.1, 1, 10, 100]) and kernel type (linear, RBF). For RF, we optimised the number of trees (range: [50, 100, 200]) and maximum depth (range: [10, 20, None]). Similar parameter grids were defined for other models, such as learning rate and number of estimators for GBC and AdB, and k values for KNN (range: [3, 5, 7]). This process resulted in the selection of hyperparameters that contributed to the high performance of the SVM model, achieving an accuracy of 97.50%, as well as robust results for other classifiers, as reported in Table 2. The use of grid search ensured that our models were finely tuned, enhancing their reliability for BC detection in clinical applications.

### 3.6 Trustworthiness Evaluation

To justify the trustworthiness of our hybrid model, we employed SHAP (SHapley Additive exPlanations) to provide interpretable explanations of the model's predictions, enhancing its reliability for clinical applications. SHAP was applied to the features extracted by the VGG‐16 model and classified by the SVM, quantifying the contribution of each feature to the prediction of IDC‐positive and IDC‐negative cases. This analysis revealed which histopathological patterns were most influential in the model's decisions, thereby improving transparency. We selected SHAP over alternatives like LIME or uncertainty estimation due to its theoretically grounded approach, which ensures consistent and fair attribution of feature importance across all data points, making it particularly suitable for our ensemble‐based framework. The SHAP analysis confirmed that the model's high accuracy (97.50%) is driven by meaningful features, reducing the risk of spurious correlations and enhancing trust in its diagnostic capabilities. By providing clear explanations of the model's behaviour, SHAP supports its adoption in clinical settings, where interpretability is essential for medical professionals to trust and validate automated predictions.

## 4 Experiments and Result Analysis

Here, we report on our analysis and experiment results for the purpose of detecting BC with our suggested method. To evaluate the efficacy of our method, we created the investigational setting and explored metrics such as F1‐score, sensitivity, specificity, and accuracy. The F1‐score provides a fair assessment, specificity evaluates the ability to identify negative cases, sensitivity assesses the capability to classify positive examples and accuracy gauges the overall quality of the detection results. These measurements gave us important information about how well our strategy worked, allowing us to assess it and compare it to other approaches already in use for BC screening.

### 4.1 Metrics of Performance Evaluation

As mentioned before, we evaluate the effectiveness of our approach by gauging its performance with several measures. These metrics are used to gauge various facets of the approach's effectiveness. These metrics are formulated as follows:

- **Confusion matrix**: The positive/negative classes that are divided into predicted and actual categories in the confusion matrix are TP, TN, FP, and FN. The numbers of correctly predicted positive, negative, and wrongly predicted cases, as well as the numbers of mistakenly anticipated positive and negative occurrences, are shown by the figures TP, TN, FN, and FP. A comprehensive assessment of the model's act is made possible by the confusion matrix, which provides details on the accurate and inaccurate predictions made for both (+ve) and (−ve) cases.
- **Accuracy**: We compute the percentage of accurately recognised samples from total samples. We applied the following equation to determine accuracy
(3) $$\mathrm{Accuracy}=\frac{\mathrm{TN}+\mathrm{TP}}{\mathrm{TP}+\mathrm{FN}+\mathrm{FP}+\mathrm{TN}}\;$$
- **Precision**: To determine how accurate positive predictions are, this statistic compares the total number of positive outcomes true and false with the ratio of accurately detected positive results. Here's the formula:
(4) $$\mathrm{Precision}=\frac{\mathrm{TP}}{\mathrm{TP}+\mathrm{FP}}$$
- **Recall**: This assesses the model's ability to identify affirmative cases. By dividing the entire amount of FN outcomes by the total number of true positive (TP) results, it is computed.
(5) $$\mathrm{Recall}\;=\frac{\mathrm{TP}}{\mathrm{TP}+\mathrm{FN}}$$
- **F1‐score**: The F1‐score, a single metric that combines recall and accuracy, offers a fair assessment of the model's performance.
(6) $$F1-\mathrm{score}=2\times \frac{\mathrm{Precision}\times \mathrm{Recall}}{\mathrm{Precision}+\mathrm{Recall}}$$
- **RSME**: RSME is the standard deviation of the errors that represented by the square root of the mean square error. Mathematical representation is as follows:
(7) $$\mathrm{RMSE}\;=\sqrt{\frac{\sum_{i=1}^{n}{\left(\mathrm{Predicted}\left(i\right)+\mathrm{Actual}\left(i\right)\right)}^{2}}{n}}$$
- **MSE**: In this procedure, the squared deviations between the expected and actual values are averaged.
(8) $$\mathrm{MSE}=\frac{\sum_{i=1}^{n}{\left(\mathrm{Predicted}\left(i\right)+\mathrm{Actual}\left(i\right)\right)}^{2}}{n}$$
- **MAE**: The squared variances between the actual and expected values are averaged in this process.
(9) $$\mathrm{MSE}=\frac{\sum_{i=1}^{n}\left|\left(\mathrm{Predicted}\left(i\right)+\mathrm{Actual}\left(i\right)\right)\right|}{n}$$
- **ROC curve and AUC**: The area under the ROC curve (AUC) is a graphical performance metric used to evaluate classification performance and measure class separability. Lower AUC values indicate poorer classification accuracy, whereas higher values indicate better predictive performance.

### 4.2 Result Analysis

The resulting study of several ML algorithms for BC diagnosis is shown in this section. To assess the consistency and dependability of the models, we performed a dependability study. The performance stability across datasets or iterations was investigated in this analysis to make sure that random fluctuations or dataset‐specific features had no impact on the outcomes. By evaluating the performance robustness and identifying any potential biases or restrictions, we were able to provide insights into the generalisation capacity of the models. We were able to decide with certainty if the models worked well in practical situations thanks to the dependability analysis. Table 3 provides a detailed evaluation of the effectiveness of various algorithms for the identification of BC based on a number of measures for evaluating the model, including accuracy, recall, F1‐score, precision, MAE, RMSE, and MSE. This table is also crucial, as it offers a comprehensive comparison of all evaluated algorithms, enabling researchers to assess their relative strengths and identify the most effective model for BC detection. The SVM is the top‐performing approach, with the highest accuracy (97.50%), precision (97.15%), recall (97.00%), and F1‐score (96.98%) of all the methods evaluated. Additionally, SVM consistently maintains the lowest error rates with an MAE of 0.03, an RMSE of 0.17, and an MSE of 0.03, demonstrating its better capacity to forecast incidences of BC with accuracy. These results indicate that SVM's high accuracy and low error rates make it particularly suitable for clinical applications, where minimising misdiagnoses can significantly improve patient outcomes by enabling timely and accurate treatment. At 82.00% accuracy, 82.18% precision, 82.00% recall, and 82.06% F1‐score, DT likewise shows low performance. DT demonstrates lower accuracy and reliability in handling complicated datasets, including an MAE of 0.18, an RMSE of 0.42, and an MSE of 0.18. Following closely behind is AdaBoost (AdB), which maintains low error values (MAE of 0.12, RMSE of 0.35, and MSE of 0.12) and has an accuracy, precision, recall, and F1‐score of 88.00%, 88.00%, 88.00%, and 88.00%, respectively. With an accuracy of 90.00% and 90.01%, respectively, KNN and GBC both perform competitively. GBC has an F1‐score of 90.40%, 90.05%, and 90.06%, respectively, and features precision and recall with error measures somewhat higher than the top performers. KNN achieves 90.00% precision, recall, and F1‐score with an MAE of 0.10, an RMSE of 0.32, and an MSE of 0.10. However, the performance levels of RF are lower. RF attains 91.02% accuracy, 92.03% precision, 91.01% recall, and an F1‐score of 91.07%. With an MAE of 0.09, an RMSE of 0.30, and an MSE of 0.09, their error measures are greater. ET has the greatest error metrics of all the methods, with an MAE of 0.10, an RMSE of 0.32, and an MSE of 0.10. It also demonstrates a slightly lower accuracy of 90.01%, with a precision of 90.79%, a recall of 90.01%, and an F1‐score of 90.07%. With an accuracy of 94.01%, a precision of 94.37%, a recall of 94.02%, and an F1‐score of 94.03%, XGB exhibits modest performance. The error metrics are MAE at 0.06, RMSE at 0.24, and MSE at 0.06. When it comes to detecting BC, the SVM algorithm is the most dependable overall. It performs exceptionally well in all evaluation metrics, which makes it a promising option. The superior performance of SVM suggests its potential to set a new standard for automated BC detection, offering a reliable tool for pathologists to enhance diagnostic precision.

**TABLE 3:** Performance evaluation of eight machine learning algorithms for breast cancer detection using multiple metrics on the IDC dataset.

Algorithm	Accuracy (%)	Precision (%)	Recall (%)	F1‐score (%)	MAE	RMSE	MSE
DT	82.00	82.18	82.00	82.06	0.18	0.42	0.18
AdB	88.00	88.00	88.00	88.00	0.12	0.35	0.12
GBC	90.00	90.40	90.00	90.06	0.10	0.32	0.10
KNN	90.00	90.00	90.00	90.00	0.10	0.32	0.10
RF	91.00	92.03	91.00	91.07	0.09	0.30	0.09
ET	90.00	90.79	90.00	90.07	0.10	0.32	0.10
XGB	94.00	94.37	94.00	94.03	0.06	0.24	0.06
SVM	97.50	97.15	97.00	96.98	0.03	0.17	0.03

To ensure the generalisability and robustness of the proposed model, external validation was conducted using two publicly available independent datasets: IDC and BreakHis [36]. The performance in Table 4 of the model across both datasets consistently demonstrated high accuracy, precision, recall, and F1‐score, validating its effectiveness in diverse clinical imaging contexts. Notably, the SVM classifier maintained superior performance across datasets, achieving accuracy rates of up to 97.50% (IDC) and 94.00% (BreakHis), confirming the model's reliability in unseen real‐world data.

**TABLE 4:** Performance evaluation of eight machine learning algorithms for breast cancer detection using multiple metrics on the BreakHis dataset.

Algorithm	Accuracy (%)	Precision (%)	Recall (%)	F1‐score (%)	MAE	RMSE	MSE
DT	70.00	71.50	70.00	69.71	0.30	0.55	0.30
AdB	86.00	85.26	83.50	82.13	0.13	0.39	0.12
GBC	89.00	89.02	89.00	89.00	0.11	0.33	0.11
KNN	76.00	82.50	76.00	75.02	0.24	0.49	0.24
RF	89.00	89.17	89.00	89.00	0.11	0.33	0.11
ET	86.00	86.59	86.00	85.98	0.14	0.37	0.14
XGB	93.00	93.02	93.00	93.00	0.07	0.26	0.07
SVM	94.00	94.00	94.00	94.00	0.06	0.24	0.06

**FIGURE 5:**
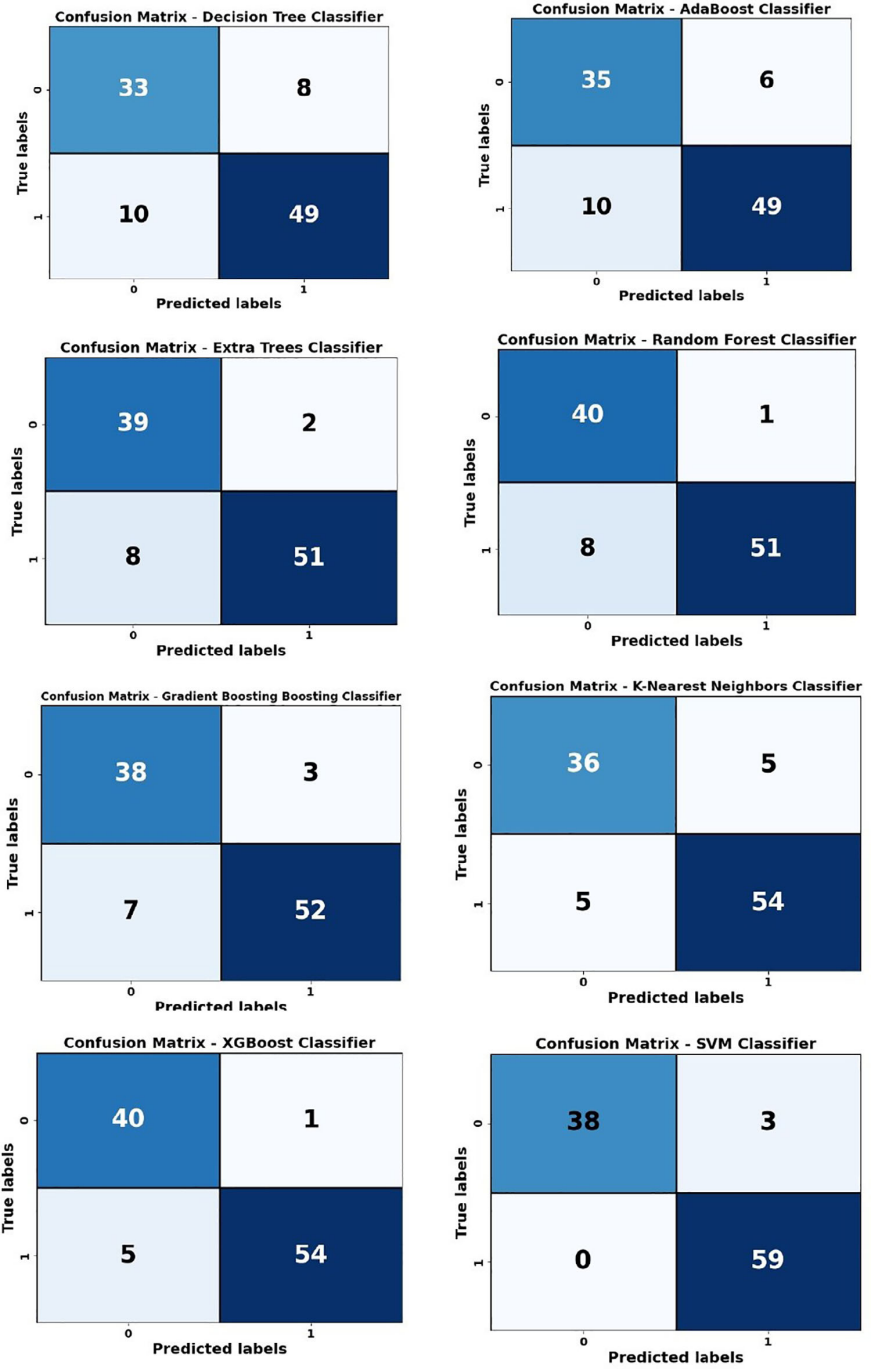
Confusion matrix for breast cancer classification.

**FIGURE 6:**
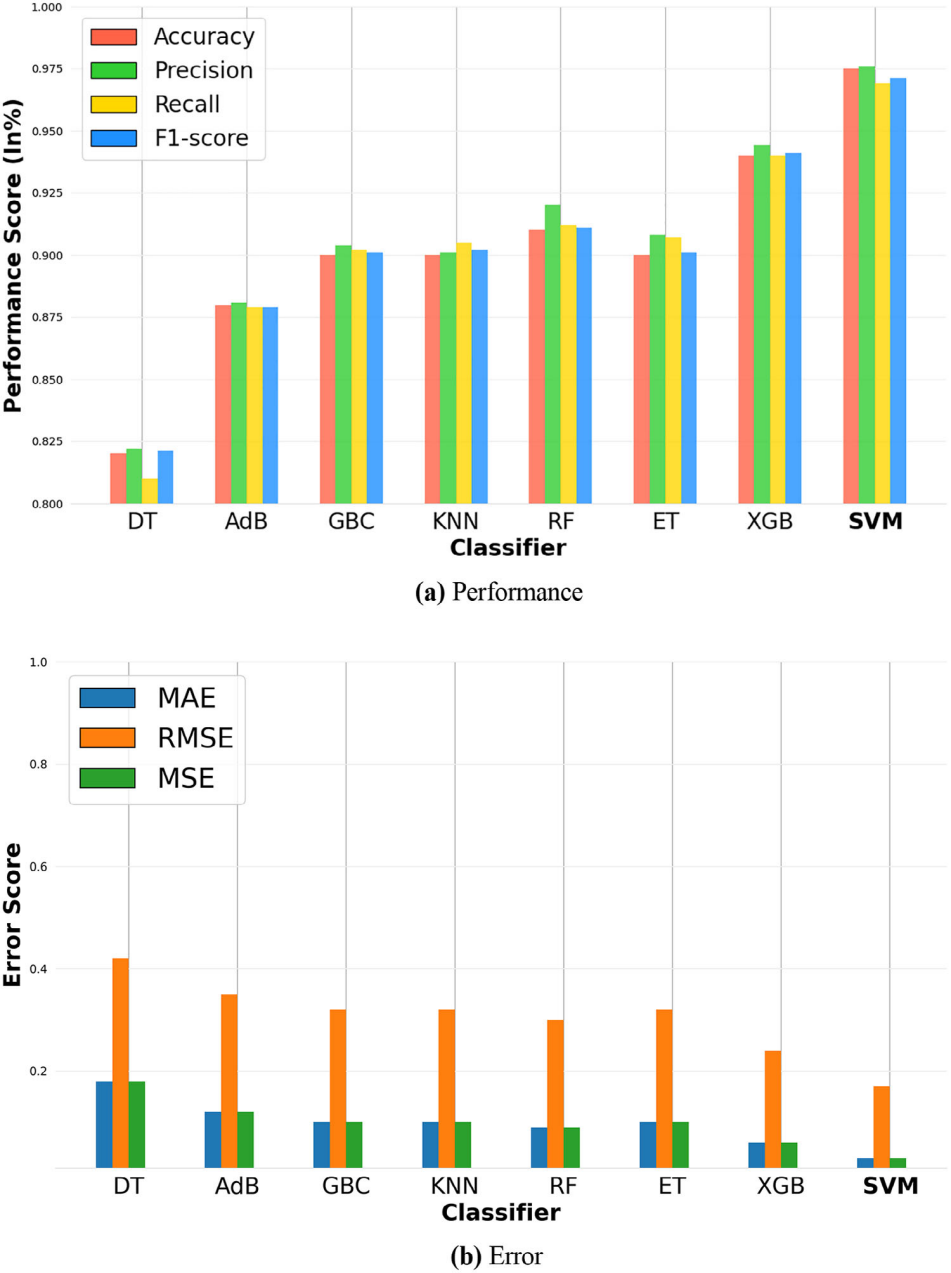
Visual evaluation of breast cancer detection performance.

The confusion matrices in Figure 5 provide important information on the accuracy and misclassification rates of many algorithms in connection to BC detection. This is also essential for visualising the classification performance of each algorithm, as it clearly illustrates the distribution of TPs, true negatives (TNs), false positives (FPs), and false negatives (FNs), aiding in the assessment of model reliability. In the DT algorithm's confusion matrix, for instance, 33 real negative instances, 8 FN cases, 49 real positive cases, and 10 bogus positive cases are shown. The convergence matrix of the SVM algorithm similarly shows that it produced 38 TN instances, 0 FP cases, 3 FN cases, and 59 TP cases. KNN generated a confusion matrix with 36 TN cases, 5 FP cases, and 5 FN cases. AdB's confusion matrix showed 50 TP cases, 6 FN cases, 10 FP cases, and 35 TN cases. The RF approach had 40 TN cases, 8 FP cases, 1 FN case, and 51 TP cases. The ET algorithm's confusion matrix showed 39 TN cases, 8 FP cases, 2 FN cases, and 51 TP cases. XGB supplied 40 TN cases, 1 FN case, 5 FP cases, and 54 TP cases. Another XGB result included 40 TN cases, 5 FP cases, 1 FN case, and 54 TP cases. The SVM algorithm demonstrates higher TP and TN cases with fewer FP and FN cases, making it more effective in identifying BC. SVM's confusion matrix had 59 TP cases, 3 FN cases, 0 FP cases, and 38 TN cases, indicating its low likelihood of misclassifying cases as FP or FN. It is more sensitive in detecting TP cases. The SVM's minimal FPs and FNs, as shown in this figure, are particularly significant in clinical settings, where reducing FNs ensures that fewer cancer cases are missed, and minimising FPs prevents unnecessary patient anxiety and follow‐up procedures. For BC screening, SVM consistently yields lower FP and FN values while achieving higher TP and TN counts. With a precision of 97.15 for the 0 class, SVM shows a low FN rate, while a recall value of 97.01 indicates a high percentage of TP cases accurately detected. The F1‐score of 96.98 reflects balanced performance. These metrics highlight SVM's ability to identify BC cases effectively, making it a reliable choice for such tasks. Additionally, its capacity to reduce FP and FN cases ensures consistent and fair classification for both positive and negative BC cases. Figure 6 represents the visual evaluation of BC detection performance. This figure also complements the numerical results by providing a visual representation of model performance across metrics, making it easier to compare the algorithms’ effectiveness and identify SVM as the standout performer.

Table 5 displays a report on the classification of BC. It provides an overview of how successfully BC is identified by ML techniques. This table is also critical for summarising the per‐class performance of the algorithms, offering insights into their ability to distinguish between IDC‐positive and IDC‐negative cases. The results for each class indicate that the SVM approach performs extraordinarily well. For the negative IDC class, the SVM approach obtains a precision of 99.01 and a recall score of 93.01. Therefore, the F1‐score, which for this class was found to be 96.06, offers a fair performance indication. The SVM method obtains a precision of 95.01 for the positive IDC class, indicating a low FN rate. A high percentage of TP instances that were accurately detected is shown by the recall value of 99.99. With an estimated F1‐score of 98.01, this class performs in a generally balanced manner. These findings demonstrate how well the SVM algorithm can identify cases of BC, which makes it a viable option for jobs involving the identification of BC. The high precision and recall for both classes, as detailed in Table 6, underscore SVM's robustness in handling imbalanced or complex histopathological data, enhancing its reliability for real‐world diagnostic applications.

**TABLE 5:** Classification performance of machine learning algorithms for IDC‐positive and IDC‐negative breast cancer classes.

	DT			AdB			GBC			KNN		
Class	Precision	Recall	F1‐score	Precision	Recall	F1‐score	Precision	Recall	F1‐score	Precision	Recall	F1‐score
0	0.77	0.80	0.79	0.85	0.85	0.85	0.84	0.93	0.88	0.88	0.88	0.88
1	0.86	0.83	0.84	0.90	0.90	0.90	0.95	0.88	0.91	0.92	0.92	0.92
Accuracy	0.82	0.82	0.82	0.88	0.88	0.88	0.90	0.90	0.90	0.90	0.90	0.90
Macro average	0.81	0.82	0.82	0.88	0.88	0.88	0.89	0.90	0.90	0.90	0.90	0.90
Weighted average	0.82	0.82	0.82	0.88	0.88	0.88	0.90	0.90	0.90	0.90	0.90	0.90

	**RF**			**ET**			**XGB**			**SVM**		
**Class**	**Precision**	**Recall**	**F1‐score**	**Precision**	**Recall**	**F1‐score**	**Precision**	**Recall**	**F1‐score**	**Precision**	**Recall**	**F1‐score**
0	0.83	0.98	0.90	0.83	0.95	0.89	0.89	0.98	0.93	0.99	0.93	0.96
1	0.98	0.86	0.92	0.96	0.86	0.91	0.98	0.92	0.95	0.95	0.99	0.98
Accuracy	0.91	0.91	0.91	0.90	0.90	0.90	0.94	0.94	0.94	0.98	0.97	0.978
Macro average	0.91	0.92	0.91	0.90	0.91	0.90	0.94	0.95	0.94	0.98	0.96	0.97
Weighted average	0.92	0.91	0.91	0.91	0.90	0.90	0.94	0.94	0.94	0.97	0.97	0.97

**TABLE 6:** Fivefold cross‐validation performance of the proposed model.

K‐fold	Accuracy (%)	Precision (%)	Recall (%)	F1‐score (%)
Fold 1	95.56	95.57	95.56	95.55
Fold 2	97.22	97.27	97.22	97.22
Fold 3	92.78	92.93	92.78	92.78
Fold 4	93.33	93.34	93.33	93.33
Fold 5	96.67	96.69	96.67	96.67
**Average**	**95.11**	**95.16**	**95.11**	**95.11**

**FIGURE 7:**
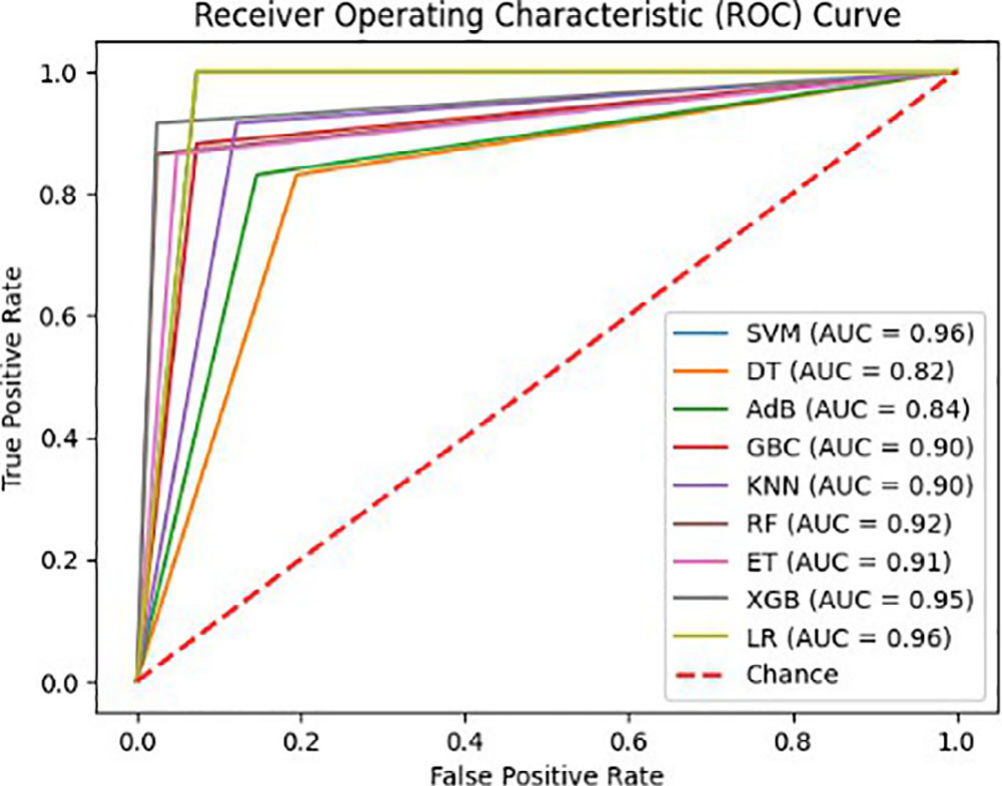
ROC curve for classifier evaluation.

As the classification report suggests, Figure 7 further shows that the SVM algorithm exhibits good recall and precision values for both positive and negative cancer cases. Its F1‐score ratings demonstrate a strong overall performance by striking a fair balance between recall and precision. Furthermore, the AUC score of 96 for the SVM algorithm is remarkable, demonstrating its strong discriminatory ability to discern between positive and negative cases. According to this study, there is a greater chance that positive cases will be given higher anticipated probabilities. This figure is also pivotal for illustrating the SVM's superior discriminative power through its high AUC score, which indicates its ability to effectively separate cancerous and non‐cancerous cases, a key factor in ensuring diagnostic reliability. The SVM algorithm offers the best precision, accuracy, F1‐score, recall, and AUC score for BC detection, according to the evaluation metrics. Collectively, these results position our SVM‐based approach as a significant advancement in automated BC detection, with the potential to improve early diagnosis rates and reduce the burden on pathologists, ultimately contributing to better patient care. The proposed model demonstrates consistent and robust performance across all folds in the fivefold cross‐validation, achieving an average accuracy of 95.11%, precision of 95.16%, recall of 95.11%, and F1‐score of 95.11%. The results provided in Table 6 indicate the model's reliability and effectiveness in BC detection.

### 4.3 Discussion

Table 7 presents a comparative discussion of various methods for detecting BC and their associated accuracy rates. Anjum et al. [12], for instance, used SVM in conjunction with HOG and Canny Edge approaches to achieve a 94.00% accuracy rate, but with lesser accuracy for noncancerous patients. Using a CNN‐based methodology [25] achieved an 86.00% accuracy rate for the identification of IDC. By using a ResNet152 model with FC layers, Kulkarni and Sundaray [28] were intelligent to achieve a 91.00% accuracy rate. In a comparison between ResNet‐50 and a self‐defined CNN model, Jin and Xie [29] found that the latter had an accuracy rate of 86.57%. Other research that used transformer‐based techniques included [10] ResNet‐50, VGG19^4^, and pre‐trained ResNet50V2 [1]. Their accuracy rates ranged from 90.20% to 96.88%. Other works, such as [30], achieved a 91.37% accuracy rate using DenseNet201, ResNet50, ResNet101, and MobileNet‐v2. Moreover, research utilising CNNs and ViTs [31] achieved an impressive 97.02% accuracy rate. Notably, among the stated methods, our suggested model, which uses SVM for classification and VGG‐16 for feature extraction, obtained the best accuracy rate of 97.50% in the identification of BC. Our model's superior accuracy aligns with the high performance of CNN and ViT‐based approaches, which also achieved near 97.00% accuracy, likely due to their robust feature extraction capabilities. However, our results surpass those of Anjum et al. [12] and Kulkarni and Sundaray [28], potentially because our hybrid approach leverages VGG‐16's deep feature extraction to capture intricate histopathological patterns, combined with SVM's effective handling of non‐linear data relationships. In contrast, our findings contradict the lower accuracy rates reported by Jin and Xie [29] and the CNN‐based method [25], which may be attributed to their reliance on less optimised architectures or smaller datasets that limited their ability to generalise across complex IDC cases. The alignment with high‐performing models like ViTs underscores the efficacy of combining DL with traditional ML, while the improvement over other methods highlights the advantage of our tailored feature extraction and classification strategy. Additionally, a recent study [36] using a CNN‐based approach achieved a 96.94% accuracy rate, closely aligning with our results. This similarity suggests that advanced neural architectures, like our VGG‐16‐based feature extraction, are highly effective for IDC detection. However, our model's slight edge may stem from the SVM's superior handling of high‐dimensional features extracted by VGG‐16, enhancing classification precision. There are various advantages to using SVM in our model for BC screening. To begin with, SVM is a support vector framework that is well known for its great speed and efficiency, which makes it ideal for processing big datasets quickly. This effectiveness makes it possible to identify and diagnose BC more quickly, which is necessary for early interference and better patient consequences. Furthermore, SVM is capable of managing intricate and non‐linear interactions in the data, allowing the model to identify minute deviations and complex patterns that might be signs of BC. Our model is more accurate and reliable when it captures complicated interactions, which lowers the possibility of FPs or FNs and guarantees high diagnostic reliability, as evidenced by the low FP and FN rates in our confusion matrices (Figure 5), which further supports its potential for clinical adoption.

**TABLE 7:** Accuracy comparison of our hybrid model with state‐of‐the‐art models.

Reference	Feature extraction	Deep learning/machine learning	Dataset	Accuracy rate
[1]	ResNet50V2	LGB	IDC	95.00%
[4]	—	ResNet‐50, VGG‐19	IDC	90.20%
[10]	—	Transformer‐based + CNN	IDC	96.88% (avg)
[12]	HOG and Canny Edge	SVM	IDC	94.00%
[25]	—	CNN	IDC	86.00%
[27]	LASSO	RF	SEER breast cancer data	90.680%
[28]	ResNet152	FC layer	IDC	91.00%
[29]	—	ResNet‐50	IDC	86.57%
[30]	—	DenseNet201, ResNet50, ResNet101, MobileNet‐v2	Histopathological images	91.37%
[31]	—	CNNs and vision transformers (ViTs)	BreakHis	97.02%
[36]	—	CNN	Histopathological images	96.94%
**Our model**	**VGG‐16**	**SVM**	**IDC**	**97.50%**

### 4.4 Limitations

Although our hybrid model yields encouraging results, a fair assessment of our results must consider its limitations. The fact that our research is limited to IDC may limit the applicability of our findings to other subtypes of BC. Additional investigation is required to evaluate its efficacy in treating various cancer types. The calibre and diversity of the training dataset affect our model's performance. Even though we used a large dataset, the model's generalisation abilities might be further improved by the availability of more diverse and vast datasets. Even though our model proves remarkable accuracy, there is still potential for improvement, mainly when noise and distortions are present in the image. Additional model optimisation and improvement may improve the model's performance in clinical settings. Specifically, the dataset's exclusive focus on IDC introduces a potential bias, as it may not adequately represent other BC subtypes, such as ILC, potentially reducing the model's effectiveness in diverse clinical scenarios. Furthermore, the reliance on a fixed patch size (50 × 50 pixels) may overlook larger contextual features in histopathological images, which could limit the model's ability to detect subtle variations in tumour morphology. The dataset, while large with 277,524 patches, is derived from only 162 whole‐mount slides, which may constrain the diversity of patient profiles and tumour characteristics, potentially affecting generalisability to broader populations. Additionally, the model's performance may be sensitive to image quality variations (e.g., noise or staining inconsistencies), which could introduce errors in real‐world settings where imaging conditions vary. These limitations suggest that while our model excels in IDC detection, its application to other subtypes or less controlled clinical environments requires further validation and dataset expansion. Notwithstanding these drawbacks, our hybrid model for detecting BC makes a significant contribution to the field and provides insightful information for precise IDC diagnosis. Like any trustworthy technique, more investigation and validation on more wide‐ranging and more varied datasets are necessary to evaluate its possible medical impact and expand its pertinency to a broader range of instances of BC.

## 5 Conclusion and Future Research

The hybrid, trustworthy model for BC diagnosis presented in this paper combines DL and ML methods. Our method takes advantage of the pre‐trained VGG‐16 TL model's DL foundation to extract intricate patterns and depictions from HPI‐based IDC images that are accessible to the general public. We proved the efficacy of our method by attaining excellent accuracy, precision, recall, and F1‐score through rigorous testing on a huge dataset of IDC. Our study addresses a number of shortcomings in previous research, which greatly advances the identification of BC. This model provides a dependable and precise method for IDC identification by combining DL and ML approaches. In the BC classification report, while ML methods increase the interpretability and simplification abilities of the model, DL integration enables the model to learn and extract significant features from complicated image data. Moreover, we assure the repeatability and accessibility of our study findings by using publicly available images of breast histopathology, mostly concentrating on IDC. Through our attention to this dataset, we offer important new information about the possibility of our method to enable accurate and early identification of IDC. Furthermore, our extensive testing yields strong proof of the resilience and excellent performance of our crossbreed model. Our approach shows that it is effective in precisely detecting IDC with an enhanced accuracy rate of 97.50%, precision of 97.15%, recall of 97.00%, F1‐score of 96.98%, and low error rates of 3%, 3%, and 17%. Furthermore, our experiment has the potential to support medical specialists in their decision‐making and eventually lead to better patient care. Then, after extracting features using a trained VGG‐16 model, we suggest that SVM is the best ML method for classifying BC based on our findings. This study significantly contributes to the field by providing a highly accurate and interpretable model for IDC detection, which enhances early diagnosis, reduces diagnostic errors, and supports pathologists in delivering timely interventions, ultimately improving patient outcomes. Additionally, our hybrid approach sets a new benchmark for automated BC detection, offering a scalable framework that can inspire further advancements in medical imaging research. Even when the hybrid model yields encouraging findings, there are a number of directions that more studies can go to improve the classification and treatment of BC. Future research should focus on integrating multi‐modal data, such as genomic profiles, mammographic images, and clinical records, to develop a more holistic diagnostic model that captures diverse aspects of BC pathology. Additionally, enhancing model robustness through advanced data augmentation techniques and transfer learning could improve performance across varied imaging conditions and patient demographics. Validating the model on datasets that include other BC subtypes, such as ILC, is critical to ensuring broader clinical applicability. Finally, exploring ensemble methods that combine multiple DL architectures with ML classifiers could further boost accuracy and generalisation, paving the way for real‐world deployment in clinical settings. *Combining data from many modes*: A more complete knowledge of BC may be obtained by combining different data sources such as genetic data, clinical data, or other imaging modalities (MRI, mammography, etc.). By combining multiple modalities, significant new insights into the biological processes that affect the onset, course, and treatment response of cancer may be obtained. *Improvement of the robustness of the model*: Further modifications to the hybrid model's design and training process can enhance its robustness to changes in patient demographics, tissue composition, and image quality. This study explored techniques to improve model performance and generalisation capabilities, including transfer learning, ensemble methods, and data augmentation.

## Author Contributions

**Md. Rashed**: contributed to conceptualization, data curation, resource management, software development, visualization, and drafted the original manuscript. **Mohammad Kamrul Hasan**: contributed to funding acquisition, software development, supervision, validation, and participated in writing, reviewing, and editing the manuscript. **Md. Imran Hossain**: contributed to conceptualization, data curation, investigation, supervision, and validation. **Md. Sarwar Hosain**: contributed to formal analysis, validation, and critical review and editing of the manuscript. **Abdul Hadi Abd Rahman**: contributed to data curation, formal analysis, funding acquisition, investigation, methodology development, and manuscript review and editing. **Shayla Islam**: contributed to data curation, formal analysis, funding acquisition, investigation, methodology, project administration, and visualization. All authors have read and approved the final manuscript.

## Funding

This work has been supported by the Universiti Kebangsaan Malaysia (UKM), Under research grant Scheme, DIP 2024‐033.

## Conflicts of Interest

The authors declare no potential conflicts of interest.

## Data Availability

The data that support the findings of this study are openly available in the Breast Histopathology Images repository at https://www.kaggle.com/datasets/paultimothymooney/breast‐histopathology‐images.
